# Post-extubation dysphagia in pediatric trauma patients: a single-center case-series study

**DOI:** 10.1038/s41598-024-54247-x

**Published:** 2024-02-12

**Authors:** Naoki Yogo, Takeru Abe, Kyoko Kano, Yuichiro Muto, Sachi Kiyonaga, Katsuki Hirai

**Affiliations:** 1 Department of Pediatric Emergency and Critical Care Medicine, Japanese Red Cross Kumamoto Hospital, 2-1-1 Ngamineminami Higashi-ku, Kumamoto, 861-8520 Japan; 2 Department of Quality and Safety in Healthcare, Yokohama City University Medical Center, 4-57 Urafunecho, Minami-ku, Yokohama, Kanagawa 232-0024 Japan; 3 Department of Rehabilitation, Japanese Red Cross Kumamoto Hospital, 2-1-1 Ngamineminami, Higashi-ku, Kumamoto, 861-8520 Japan

**Keywords:** Paediatric research, Brain injuries, Paediatric research

## Abstract

We aimed to investigate whether ventilator support time influences the occurrence of dysphagia in pediatric trauma patients. This case-series study was conducted in a single pediatric emergency and critical care center from April 2012 to March 2022. Trauma patients aged < 16 years who underwent tracheal intubation were divided into two groups based on the occurrence of dysphagia within 72 h after extubation, and their data were analyzed. Tracheal intubation was performed in 75 pediatric trauma patients, and 53 of them were included in the analysis. A total of 22 patients had post-extubation dysphagia and head trauma. The dysphagia group tended to have more severe head injuries (Abbreviated Injury Scale (AIS) 4 [4–5] vs. 4 [0–4]; *p* < 0.05), a longer ventilator support time (7 days [4–11] vs. 1 day [1–2.5]; *p* < 0.05), and a longer length of hospital stay (27 days [18.0–40.3] vs. 11 days [10.0–21.0]; *p* < 0.05). Severe head trauma and a long duration of tracheal intubation may be risk factors for dysphagia in pediatric trauma patients. Therefore, early recognition of these risk factors could assist in treatment planning for speech-language pathologist intervention and nutritional routes of administration.

## Introduction

Post-extubation dysphagia is a swallowing disorder that occurs predominantly in intensive care units of both adults and children. The frequency of post-extubation dysphagia in adults ranges from 3 to 62%^1^. In children, the rate of dysphagia has been reported to be 29%^2^, although only a few studies have investigated this observation. Dysphagia increases the risk of aspiration pneumonia, leading to longer hospital stays and increased mortality^3^. At present, a known risk factor for post-extubation dysphagia is the duration of tracheal intubation or ventilatory management; the longer the duration, the higher the probability of occurrence^1,3–5^. The mechanisms underlying dysphagia can be divided into mechanical and cognitive factors. The mechanical factors include pharyngolaryngeal injury from tracheal tubes, edema, and sensory disturbances, while cognitive factors include head trauma, stroke, and delirium, leading to poor coordination of swallowing movements^6^. Post-extubation dysphagia has been well-studied in adults with trauma^5^ but not in pediatric trauma patients. Children differ from adults considerably in terms of both mechanical and cognitive factors. Owing to the increasing cases of head injuries in trauma^7^, children may be at a higher risk of developing dysphagia due to having an immature swallowing function compared to that of adults^8^. To the best of our knowledge, risk factors for dysphagia in pediatric patients with trauma have not yet been identified. Hence, this study aimed to investigate whether ventilator support time or other factors influenced the occurrence of dysphagia in pediatric trauma patients. It is important to note that the number of pediatric patients in this study was small and it was difficult to accumulate cases; therefore, we considered it necessary to accumulate findings through a case series.

## Methods

### Study design and population

Given the limited occurrence of pediatric trauma cases with dysphagia, it is difficult to accumulate cases; therefore, we decided to accumulate findings through a case-series approach in this study. This case-series study was performed at a pediatric emergency center, a tertiary-care facility approved by the Ministry of Health, Labor, and Welfare. Patients were enrolled from April 1, 2012, to March 31, 2022. We included patients with trauma aged < 16 years who were intubated, including patients with short-term (< 48 h) ventilatory management. We excluded the following patients: those who died, had a tracheostomy, were evaluated by a speech-language pathologist or nurse for more than 72 h after extubation, or were originally on home ventilator management. Swallowing function was assessed by nurses with more than 3 years of experience in the pediatric intensive care unit (PICU). The nurses conducted the evaluation based on a feeding and swallowing training protocol developed by a multidisciplinary team at our facility. In principle, the nurses made the final judgment based on the presence or absence of dysphagia. However, in case of doubt, the decision was made after consultation with speech-language pathologists. In this study, speech-language pathologists did not perform videofluoroscopic swallow studies or fiberoptic endoscopic swallow evaluations because of the acute phase of swallowing.

### Ethics approval and consent to participate

The study protocol was approved by the Ethics Committee of the Japanese Red Cross Kumamoto Hospital (approval number 510), which waived the requirement for obtaining informed consent. All the methods and procedures carried out in this study were in accordance with relevant guidelines and regulations.

### Data collection

Patient data were obtained from medical records. The demographic and clinical variables collected included age, sex, weight, PICU admission diagnosis, Abbreviated Injury Scale (AIS), and Injury Severity Score (ISS). The primary outcome measure was the presence or absence of signs of dysphagia on the first feeding following extubation, such as feeding-related coughing, choking, wet and gurgling voice, breathing quality, and/or bradycardia with desaturation. The first evaluation method was the modified water swallowing test^9^, as per our protocol. First, the patients were instructed to drink 1 mL of cold water to ensure there was no choking, wet hoarseness, or respiratory changes. If the patients swallowed three times without problems, they proceeded to the trial feeding phase with swallowing jellies. At this point, if the patients could chew and swallow adequately without experiencing choking, wet hoarseness, or respiratory changes, they were considered to have no dysphagia in this study. In the case of infants, a nipple was introduced into the mouth to confirm that sucking was sufficient. Initially, 1-mL milk was provided via a bottle three times to confirm the absence of choking, wet hoarseness, or respiratory changes. Subsequently, 10-mL milk was provided via a bottle; if no problems arose, the patient was considered free of dysphagia. The secondary analysis examined the occurrence of post-extubation dysphagia in terms of the following patient outcomes: length of PICU stay, length of hospital stay, time elapsed from extubation to initiation of oral intake, time to reach total oral intake, enteral feeding at hospital discharge, post-extubation pneumonia, and reintubation.

### Statistical analysis

Categorical data (sex, primary diagnosis, age 0–24 months vs. 25 months and above, presence of post-extubation dysphagia, need for emergency intubation, complex chronic conditions, operation, prior use of vasopressors, neuromuscular blockade, benzodiazepine, barbiturate, and opioids) were reported as frequencies and percentages and presented as median and interquartile range.

Comparison between the control and post-extubation dysphagia groups was based on the Mann–Whitney U test for continuous variables and Fisher’s exact test for categorical variables. All statistical tests were two-tailed, and *p* < 0.05 was considered significant. Data were analyzed using EZR (Saitama Medical Center, Jichi Medical University, Saitama, Japan), a graphical user interface for R (The R Foundation for Statistical Computing, Vienna, Austria). In particular, it is a modified version of the R commander designed to add statistical functions frequently used in biostatistics.

## Results

### Descriptive profile of the patients

**Figure 1:**
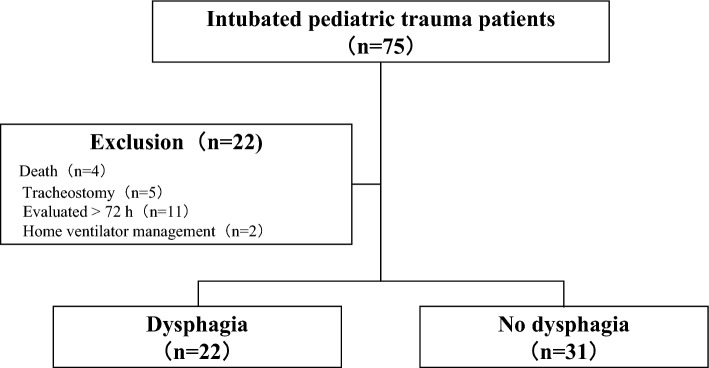
Flow diagram illustrating patient selection.

During the 10-year study period, 75 pediatric trauma patients required endotracheal intubation (Fig. 1). After applying the inclusion and exclusion criteria, we included 53 patients in the analysis, and 22 of them experienced post-extubation dysphagia (Tables 1, 2). The median age was 58 months (15–84), and 62.3% of the patients were boys. The median number of days these patients received mechanical ventilation, stayed in the PICU, and stayed in the hospital were 4 (1–7), 6 (3–12), and 17 (11–29), respectively. The proportion of patients with a primary diagnosis of traumatic brain injury (TBI) was 83.0% (44/53), and the median head/neck AIS score was 4 (3–4). Emergency intubation was performed in 60.4% (32/53) of patients, and surgery was performed in 77.4% (41/53) of patients. Approximately 9.4% of the patients were administered vasopressors, 11.3% were administered neuromuscular blockade drugs, 77.4% were administered benzodiazepine, 100% were administered opioids, and 28.3% were administered barbiturates. The median probability of survival was 97.6%.

**Table 1:** Bivariate analysis of patient characteristics with and without post-extubation dysphagia.

Characteristics	Full cohort (n = 53)	Dysphagia (n = 22)	No dysphagia (n = 31)	*p* value
Age, months (median (IQR)	58 (15–84)	57 (11–82.8)	58 (25–86.5)	1
Age (categorical) (months)				0.55
0–24	16 (30.2%)	8 (36.4%)	8 (25.8%)	
25+o	37 (69.8%)	14 (63.6%)	23 (74.2%)	
Gender				1
Male	33 (62.3%)	14 (63.6%)	19 (61.3%)	
Emergent intubation				< 0.05
Yes	32 (60.4%)	18 (81.8%)	14 (45.2%)	
Primary diagnosis (%)				
Traumatic brain injury	44 (83.0%)	22 (100%)	22 (71%)	< 0.05
Weight, kg, median (IQR)	16 (10–24)	16 (8.6–24.3)	16 (12–22)	0.91
Duration of intubation, day, median (IQR)	4 (1–7)	7 (4–11)	1 (1–2.5)	< 0.05
Complex chronic conditions	5 (9.4%)	4 (18.2%)	1 (3.2%)	0.15
ISS, median (IQR)	16 (16–22)	16 (16–26)	16 (16–18.5)	0.28
Head/neck AIS, median (IQR)	4 (3–4)	4 (4–5)	4 (0–4)	< 0.05
Ps, %	97.6 (90.7–99.0)	92.2 (80.0–95.4)	98.5 (97.7–99.0)	< 0.05
Operation	42 (77.4%)	18 (81.8%)	24 (77.4%)	0.75
Prior use of vasopressors	5 (9.4%)	4 (18.2%)	1 (3.2%)	0.15
Prior use of neuromuscular blockade	6 (11.3%)	5 (22.7%)	1 (3.2%)	0.07
Prior use of benzodiazepine	41 (77.4%)	13 (59.1%)	28 (90.3%)	< 0.05
Prior use of opioids	53 (100%)	22 (100%)	31 (100%)	–
Prior use of barbiturate	15 (28.3%)	12 (54.5%)	3 (9.7%)	< 0.05

*AIS* abbreviated injury scale, *ISS* injury severity score, *IQR* interquartile range, *Ps* probability of survival.

**Table 2:** Common causes of traumatic brain injury in patients with dysphagia.

No	Age (month)	Sex	Diagnosis	Type of neurological surgery	Alternative means of nutrition at discharge	Hospital disposition
1	152	M	SDH, EDH, CI	Craniotomy for removal of hematoma, decompressive craniectomy, ICP	–	Rehab/nursing facility
2	3	M	SDH, BC, SF		–	Home
3	42	M	DAI, SAH	ICP	–	Rehab/nursing facility
4	6	F	CP, SAH, IVH, SF	Duraplasty	–	Home
5	6	F	EDH	Craniotomy for removal of hematoma	–	Home
6	20	F	SDH, SF	ICP	–	Home
7	60	F	SF, facial bone fracture		–	Home
8	71	F	DAI	Craniotomy for removal of hematoma, cranioplasty	–	Rehab/nursing facility
9	156	M	BC, SF, cervical fracture, facial bone fracture		–	Home
10	77	M	DAI, EDH, SF		–	Other
11	84	M	DAI, pelvic fracture		–	Rehab/nursing facility
12	111	M	DAI, BC, SF, facial bone fracture	ICP	–	Rehab/nursing facility
13	175	M	SAH, BC, SF	Craniotomy for removal of hematoma, decompressive craniectomy	Nasogastric tube	Rehab/nursing facility
14	4	M	EDH	Burr-hole evacuation, ICP	Gastrostomy	Rehab/nursing facility
15	54	M	DAI, facial bone fracture		Nasogastric tube	Rehab/nursing facility
16	164	M	SDH, EDH, BC, clavicle fracture	Craniotomy for removal of hematoma	–	Rehab/nursing facility
17	1	F	SDH	Burr-hole evacuation, ICP	–	Other
18	24	F	SDH, BC	Craniotomy for removal of hematoma, ventricular drainage	–	Home
19	8	F	SDH, SF	Burr-hole evacuation, ICP	Nasogastric tube	Rehab/nursing facility
20	40	M	SAH, BC, SF, facial bone fracture, lung contusion		–	Rehab/nursing facility
21	67	M	SAH, degloving injury		–	Rehab/nursing facility
22	79	M	SAH, CH, BC	Ventricular drainage	–	Rehab/nursing facility

*BC* brain contusion, *CH* cerebral haemorrhage, *CI* cerebral infarction, *CP* cerebral prolapse, *DAI* diffuse axonal injury, *EDH* epidural haemorrhage, *ICP* intracranial pressure, *IVH* intraventricular haemorrhage, *SAH* subarachnoid haemorrhage, *SDH* subdural haemorrhage, *SF* skull fracture.

### Characteristics and association between the different variables

The group with dysphagia had a higher frequency of emergent intubation than the group without dysphagia (81.8% [18/22] vs. 45.2% [14/31]; *p* < 0.05), and all patients with dysphagia had TBI. Although there was no difference in the ISS between the two groups, the AIS differed, with more severe head and facial injuries occurring in the group with dysphagia (4 [4, 5] vs. 4 [0–4]; *p* < 0.05). The median probability of survival was lower in the dysphagia group than in the group without dysphagia (92.2% [80.0–95.4] vs. 98.5% [97.7–99.0]; *p* < 0.05). The duration of tracheal intubation was significantly longer in the dysphagia group than in the group with no dysphagia (7 days [4–11] vs. 1 day [1–2.5]; *p* < 0.05). The history of benzodiazepine and barbiturate use differed between the groups, with the dysphagia group using barbiturates more frequently and the group without dysphagia using midazolam more frequently. In the dysphagia group, 63.6% (14/22) of patients underwent neurological surgery, and 32% (7/22) of patients were directly discharged to their homes.

The medan time from extubation to first oral intake differed between the two groups: 2 days in the group with dysphagia and 1 day in the group without dysphagia (Table 3). Similarly, the median time to full oral intake without the need for combined intravenous or tube feeding differed between the group with dysphagia and that without dysphagia, which were 12 and 3 days, respectively. Four patients in the dysphagia group required tube feeding at the time of discharge. The median length of stay in the PICU was 15 days in the group with dysphagia and 3 days in the group without dysphagia. The median length of hospital stay was 27 days in the group with dysphagia and 11 days in the group without dysphagia. In summary, patients with dysphagia showed a trend towards longer PICU stays.

**Table 3:** Bivariate analysis of patient outcomes.

Outcome	Full cohort (n = 53)	Dysphagia (n = 22)	No dysphagia (n = 31)	*p* value
Time elapsed from extubation to initiate oral intake	1 (0.3–2)	2 (1–5)	1 (0–1)	< 0.05
Time to reach total oral intake	5 (2–11)	12.5 (8.3–20.0)	3.0 (2.0–5.0)	< 0.05
Enteral feeding at hospital discharge	4 (7.5%)	4 (18.2%)	0 (0%)	< 0.05
Pneumonia postextubation	1 (1.9%)	1 (4.5%)	0 (0%)	0.42
Length of PICU stay	6 (3–12)	14.5 (6.3–21.8)	3.0 (2.0–6.0)	< 0.05
Length of hospital day	17 (11–29)	27 (18.0–40.3)	11 (10.0–21.0)	< 0.05
Reintubation	1 (1.9%)	1 (4.5%)	0 (0%)	0.42

*PICU* paediatric intensive care unit.

## Discussion

In this study, we investigated whether the duration of ventilation was a risk factor for post-extubation dysphagia in pediatric trauma patients, as has been shown in adults. To the best of our knowledge, this is the first study to identify duration on ventilator and the severity of a head injury as risk factors for dysphagia in pediatric trauma patients. Similar to previous reports on post-extubation dysphagia in adults^3–5^, pediatric patients with dysphagia had a longer duration of tracheal intubation. In this study, the frequency of severe head trauma was higher in patients with dysphagia than in those without dysphagia. This suggests that in pediatric trauma patients, the presence and severity of head trauma, as well as the duration of tracheal intubation, are associated with the occurrence of dysphagia.

The duration of tracheal intubation is a risk factor for post-extubation dysphagia. Macht et al.^4^ reported that ventilatory management for > 7 days was associated with dysphagia. In trauma patients, ventilatory management > 2 days is considered a risk factor for post-extubation dysphagia^5^. Furthermore, dysphagia is associated with a longer hospital stay^2–4,10^, as observed in this study. The median duration of tracheal intubation in patients with dysphagia was 7 days, and the length of stay in the PICU and hospitalization tended to be significantly longer than that in patients without dysphagia. Several probable reasons have been proposed for the occurrence of dysphagia in the intensive care unit^6,11^, namely oropharyngeal and laryngeal trauma, neuromuscular weakness, reduced laryngeal sensation, altered sensorium, gastroesophageal reflux, dyssynchronous breathing, and swallowing. Oropharyngeal and laryngeal trauma is caused by tracheal tube compression injury and granulation, leading to dysphagia. Neuromuscular weakness is associated with dysphagia owing to a decrease in muscle strength related to swallowing caused by long-term tracheal intubation management. A reduced laryngeal sensation is caused by polyneuropathy or local edema associated with a critical illness. Altered sensorium may be due to head trauma, cerebrovascular disease, delirium, or drug effects. Gastroesophageal reflux may occur due to positional problems or deep sedation. Dyssynchronous breathing and swallowing occur when breathing conditions affect the swallowing process. Of these six mechanisms, the first four are considered risk factors applicable to our study population.

Our results show the necessity of focusing on the presence or absence of head trauma and the severity of head injuries in areas where there is a significant difference in the outcome. In children, the head accounts for a large proportion of the total body mass; hence, the frequency of single head injuries is high in early childhood^12^. Therefore, head trauma is said to be more common in pediatric patients than in adults, and head trauma in pediatric trauma patients should be recognized as a risk factor for dysphagia. According to Morgan et al.^10,13^, dysphagia occurred in 3.8–5.3% of pediatric patients with head injuries. The frequency of dysphagia also reportedly increased with increasing severity of the injury, with 68–76% of patients with severe TBI reported to have dysphagia^14^. These results are similar to those of our study, and it should be recognized that patients with head trauma and severe injury have an increased probability of experiencing dysphagia. In a comparison of patients with head injury, the ventilator support time was 3–9 times longer in the group with dysphagia than in the group without dysphagia, ranging from 5.2 to 9 days. Similarly, the group with dysphagia required longer hospitalization (27–50.6 days, 9–16 times longer) and a longer time to return to normal oral intake (19–35.4 days, 16–19 times longer) than that for the group without dysphagia^10,13,14^. The results of these studies are similar to those of our study.

One significant difference in our study compared to previous studies was the difference in the use of sedative drugs. In cases of suspected increased intracranial pressure, we used barbiturate (thiamylal) in addition to hypertonic saline in our hospital because barbiturate is a second- or third-line treatment for hyper intracranial pressure^15^. However, it has been suggested that early administration of barbiturate may increase mortality and worsen brain tissue oxygenation, leading to damage^16,17^. The use of midazolam is also avoided in intensive care of adults as it increases the risk of delirium^18–20^, although the use of barbiturate may be more involved in dysphagia than does the use of midazolam. Unlike adults, the number of sedatives available for use in children is limited, with midazolam being the main sedative used.

Our study has some limitations. First, the study was a single-center study with a small sample size, which may have affected the accuracy of results and introduced bias. Second, because patients with severe head trauma are naturally on ventilation for longer periods, the relationship between each of these factors as direct risk factors for dysphagia was not fully investigated. Finally, this study lacked objective indicators for the presence of dysphagia. Established methods for assessing dysphagia include a video fluoroscopic swallow study and a functional oral intake scale in children^21,22^; it would have been beneficial if these assessments had been performed in our study.

## Conclusions

In this study, the duration of tracheal intubation was shown to be associated with the occurrence of dysphagia in pediatric trauma patients, in agreement with previous findings. Furthermore, the severity of head injuries was also shown to be a possible risk factor for dysphagia. Pediatric trauma patients with dysphagia tend to have longer PICU stays and overall length of hospital stay, requiring intensive therapeutic management. Early recognition of these risk factors may help in treatment planning for speech-language pathologist interventions and nutritional route of administration, leading to shorter hospital stays.

## Data Availability

The datasets used and/or analyzed during the current study are available from the corresponding author on reasonable request.

## Abbreviations

AIS: Abbreviated Injury Scale
ISS: Injury Severity Score
PICU: Pediatric intensive care unit
TBI: Traumatic brain injury
